# Synthesis and Catalytic Application of Two Mononuclear Complexes Bearing Diethylenetriamine Derivative Ligand

**DOI:** 10.3390/molecules25092101

**Published:** 2020-04-30

**Authors:** Chao Liu, Zhao Yang, Hao Guo, Yu-Cai Zhao

**Affiliations:** School of Chemistry and Chemical Engineering, Suzhou University, Suzhou 234000, China; hgyangzhao@stu.ahszu.edu.cn (Z.Y.); hgguohao@stu.ahszu.edu.cn (H.G.); zhoufengmei@ahszu.edu.cn (Y.-C.Z.)

**Keywords:** diethylenetriamine, complex, crystal structure, catalytic activity, catalytic application

## Abstract

Two mononuclear zero-dimensional Ni(II) and Zn(II) complexes bearing diethylenetriamine derivative ligand, namely [NiL(CH_3_COO)_2_(H_2_O)] (**1**) and [ZnL(CH_3_COO)_2_] (**2**) [L = N, N’-bis(2-hydroxybenzyl)diethylenetriamine], were synthesized under reflux conditions. The molecular composition and structure of the complexes were identified by IR, PXRD, elemental analyses, and single crystal X-ray diffraction. Complex **1** belongs to a monoclinic crystal system with the *P*2_1_/*n* space group, and Complex **2** belongs to a monoclinic crystal system with the *C*2/*c* space group. The Henry reaction of nitromethane with aromatic aldehydes was explored with Complexes **1** and **2** as the catalyst. Results from the catalytic reaction revealed that the complexes displayed excellent catalytic activities under the optimized conditions and that the substrate scope of aromatic aldehydes could be extended to a certain extent. In addition, the possible catalytic mechanism of the Henry reaction was also deduced.

## 1. Introduction

Due to their special structures with difunctional group, β-nitroalcohols are very important and valuable chemical intermediates [1,2], which can be used in the synthesis of various biologically active derivatives, including amino alcohols [3], nitroalkenes [4], nitroketones, and so on [5,6]. The β-nitroalcohol product can be obtained by the Henry reaction, a very classical and applicable C-C bond formation reaction. The most common method of synthesizing β-nitroalcohols is the nucleophilic addition reaction of nitroalkane with the carbonyl compounds in the presence of catalysts, such as organic small molecules [7,8], ionic liquids [9,10], and metal complexes [11,12]. Hence, an enormous amount of effort has been devoted to the research and development of economic and efficient catalysts for this reaction.

In the past few decades, transition metal-based catalysts, such as Cu(II), Co(III), Cr(III), and Cd(II) complexes, have been synthesized and applied to catalyze the Henry reaction [13,14,15,16], with varying degrees of success. Moreover, interest in the area of rare earth metal-based catalysts has been enhanced considerably, and a variety of rare earth metal complexes has been reported to promote the Henry reaction with good catalytic activities [17]. However, some of the catalytic systems have certain limitations such as high catalyst loading, narrow substrate range, serious pollution, and so on. Therefore, there is a bright future for the development of efficient and environmentally friendly catalysts for the Henry reaction. Ni(II) and Zn(II) complexes, two kinds of typical transition metal complexes, have attracted considerable attention due to their encouraging characteristics and broad application prospects for organic synthesis as catalysts [18,19,20,21]. In recent years, the research on and exploration of their catalytic activities in the Henry reaction have been a hotspot [22,23,24].

Efforts from our group and other research groups have been made to obtain some novel nickel and zinc complexes with excellent catalytic activities in the Henry reaction. To our satisfaction, some preliminary research results have been obtained and reported successively [22,23,24,25,26]. As a continuation of the exploratory research, we herein designed and synthesized two novel mononuclear complexes [NiL(CH_3_COO)_2_(H_2_O)] and [ZnL(CH_3_COO)_2_] bearing the N, N’-bis(2-hydroxybenzyl)diethylenetriamine ligand. Furthermore, their catalytic activities in the Henry reaction of nitromethane with aromatic aldehydes were tested, and the possible reaction mechanism was also deduced.

## 2. Results and Discussion

### 2.1. Synthesis and IR Spectra

**Scheme 1:**
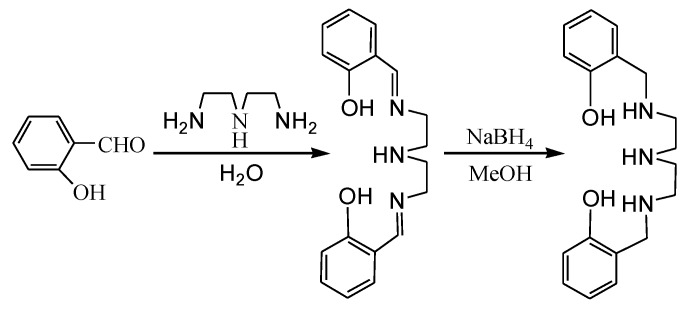
Synthesis of the ligand.

The ligand N, N’-bis(2-hydroxybenzyl)diethylenetriamine was prepared by the one pot method in two steps (Scheme 1). The reaction of diethylenetriamine with two equiv. of salicylaldehyde in water at room temperature gave the bis-Schiff base. The bis-Schiff base was then reduced by NaBH_4_ in methanol to afford the ligand in an 83% yield. The ligand is a colorless oily liquid and possesses five potential coordination sites, which can coordinate to metal atoms in various modes.

The reactions of the ligand with one equiv. of Ni(OAc)_2_ × 4H_2_O and one equiv. of Zn(OAc)_2_ × 2H_2_O in ethanol afforded two mononuclear zero-dimensional complexes [ML(CH_3_COO)_2_(H_2_O)_n_] (n = 1, M = Ni; n = 0, M = Zn), respectively. Although the central metal cations of the complexes are coordinated to the same ligand, they have different coordination modes and geometrical configurations. The complexes are soluble in some alcohol solvents such as methanol, ethanol, and isopropanol, but only slightly soluble in dichloromethane and n-hexane. They have good tolerance for moisture and air, and their crystal structures were fully authenticated by single crystal X-ray diffraction. Because of the stretching vibration of C=O in the acetate ion, a protruding infrared absorption peak was observed at 1596 cm^−1^ for Complex **1** and 1574 cm^−1^ for Complex **2**. Complex **1** exhibited medium intensity characteristic bands at 706 cm^−1^ and at 679 cm^−1^, which were assigned to the stretching vibrations of Ni―O and Ni―N, respectively. For the stretching vibrations of Zn―O and Zn―N in Complex **2**, their characteristic bands were observed at 747 cm^−1^ and 676 cm^−1^, respectively.

### 2.2. Structure Description of Complex ***1***

**Figure 1:**
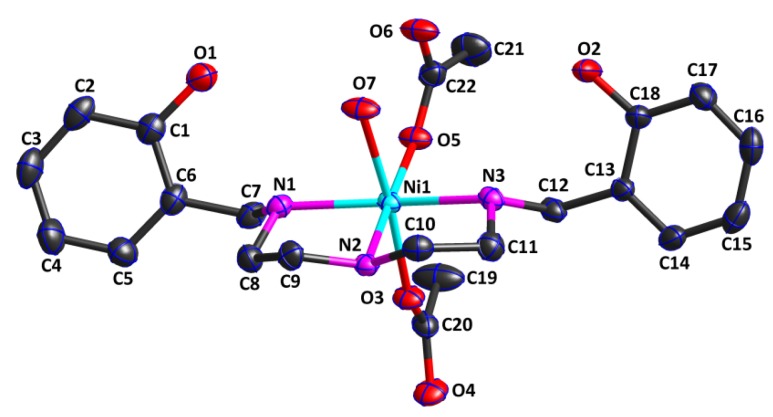
Crystal structure of Complex **1**, showing the 30% probability level of thermal ellipsoids (all H atoms are omitted for clarity).

The crystal structure analysis revealed that Complex **1** was a mononuclear zero-dimensional molecule and belonged to the monoclinic crystal system with the *P*2_1_/*n* space group. Figure 1 shows that the asymmetric unit of Complex **1** consisted of one water molecule, one ligand, two acetate anions, and one Ni(II) cation. The Ni(II) cation was hexa-coordinated by three nitrogen atoms (N1, N2, and N3) from the ligand in an η^3^ mode, one oxygen atom (O7) from one water molecule in an η^1^ mode, and two oxygen atom (O3 and O5) from two different acetates in an η^1^ mode, leading to an octahedral NiO_3_N_3_ coordination geometry. Interestingly, the oxygen atom of the phenolic hydroxyl group from the ligand did not coordinate to the Ni(II) cation, while the nitrogen atom from the ligand, with weaker coordination ability, coordinated to the Ni(II) cation. Two coordination oxygen atoms (O3 and O7) were situated in the axial position. The Ni1—O7 bond of 2.175(2) Å was longer than the Ni1—O3 bond of 2.074(2) Å, indicating that the strength of the Ni1—O3 bond was slightly stronger than that of the Ni1—O7 bond. The bond angle O3—Ni1—O7 was 173.65(10)°, deviating from 180°, indicating that the octahedral coordination geometry was slightly distorted. The four coordination atoms (N1, N2, N3, and O5) together with the central Ni(II) cation constituted the equatorial plane of the geometry structure. The sum of the bond angles N1—Ni1—N2 of 83.89(10)°, N2—Ni1—N3 of 83.30(10)°, O5—Ni1—N3 of 94.63(10)°, and O5—Ni1—N1 of 97.95(10)° around the Ni cation was 359.77°, nearly equal to 360°, which further confirmed that the equatorial plane was almost standard. The Ni1—O5 bond length was 2.031(3) Å, while the Ni1—N bond distances ranged from 2.065(3) Å to 2.121(3) Å, which were similar to those in the reported literature [27,28].

**Figure 2:**
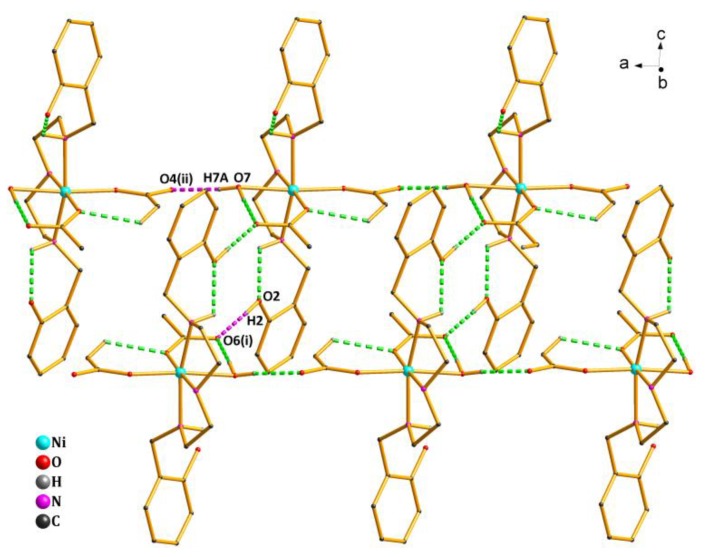
1D ladder-like chain structure of Complex **1** via hydrogen bonds along the a axis. Symmetry codes: (i) 1 − x,1 − y,1-z; (ii) 1 + x,y,z.

As shown in Figure 2, there existed four types of intramolecular hydrogen bonds and two types of intermolecular hydrogen bonds in the crystal structure of Complex **1** (Table S3). The oxygen atom (O7) from the coordinated water molecule acted as the H-donor to the oxygen atom (O4(ii)) from the acetate, forming the hydrogen bond O7—H7A…O4(ii) of 1.97 Å. The oxygen atom (O6(i)) from another acetate acted as the H-acceptor to the oxygen atom (O2) from the phenolic hydroxyl group, forming the hydrogen bond O2—H2…O6(i) of 1.82 Å. By the intermolecular hydrogen bonds quoted above, the asymmetric units of Complex **1** were further linked into an infinite 1D ladder-like chain structure along the a axis.

### 2.3. Structure Description of Complex ***2***

**Figure 3:**
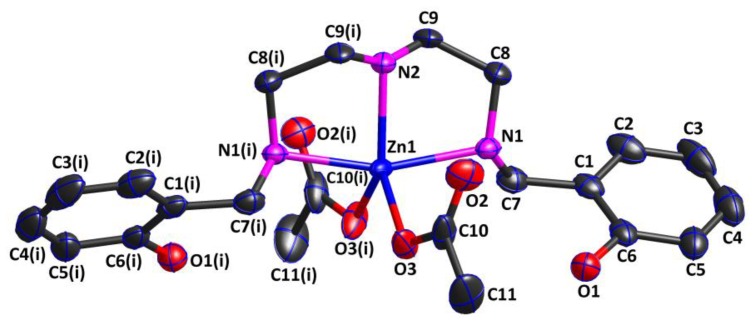
Crystal structure of Complex **2**, showing the 30% probability level of thermal ellipsoids (all H atoms are omitted for clarity). Symmetry code: (i) 1 − x,y,1/2 − z.

Complex **2** was also a mononuclear zero-dimensional molecule and belonged to the monoclinic crystal system with the *C*2/*c* space group. As shown in Figure 3, the asymmetric unit of Complex **2** consisted of one acetate, half of the Zn(II) cation, and half of the ligand. The Zn(II) cation was five-coordinated by two oxygen atoms (O3 and O3(i)) from two different acetates in an η^1^ mode and three nitrogen atoms (N1, N2, and N1(i)) from the ligand in an η^3^ mode, presenting a trigonal bipyramidal ZnO_2_N_3_ coordination geometry. Similar to the Ni(II) cation in Complex **1**, the oxygen atom of the phenolic hydroxyl group from the ligand did not coordinate to the Zn(II) cation, while the nitrogen atom from the ligand did. According to the reported literature [29], the Zn(II) complex with the same ligand also exhibited a trigonal bipyramidal ZnO_2_N_3_ coordination geometry, but the oxygen atoms coordinated to the Zn(II) cation were the oxygen atoms of the phenolic hydroxyl groups, not those of the acetates. Additionally, the ligand coordinated to the Zn(II) cation in the chelating manner, generating two five-membered chelating rings, which were both nearly planar. The three coordination atoms (O3, O3(i), and N2) together with the central Zn(II) cation defined the equatorial plane of the geometry structure, while N1 and N1(i) atoms were situated in the axial position. The bond angles O3—Zn1—N2 of 129.73(6)°, O3—Zn1—O3(i) of 100.55(9)°, and O3(i)—Zn1—N2 of 129.73(6)° added up to 360.01°, approximately equal to 360°, indicating that the four atoms were in an almost perfect plane. The bond angle N1—Zn1—N1(i) was 161.74(7)°, which seriously deviated from 180°, indicating that the Zn1 cation was in a distorted trigonal bipyramidal environment. Furthermore, the two Zn1—O bond lengths were both 1.974(2) Å, while the Zn1—N bond distances fell in the ranges of 2.198(2) and 2.092(3)Å, which were comparable to those of the related Zn(II) complexes [30,31].

**Figure 4:**
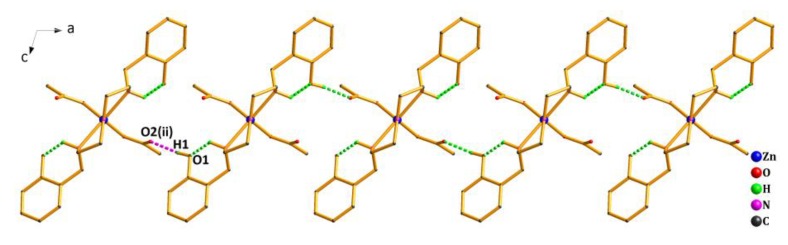
1D hand-in-hand-like chain structure of Complex **2** via hydrogen bonds along the a axis. Symmetry code: (ii) 1/2 − x, −1/2 + y,1/2 − z.

**Figure 5:**
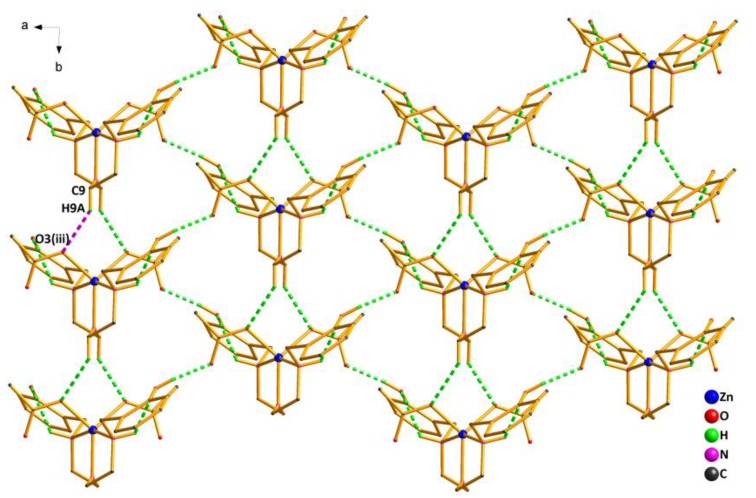
2D wave-like network structure of Complex **2** via hydrogen bonds along the ab plane. Symmetry code: (iii) 1 − x,1 + y,1/2 − z.

As shown in Figure 4, the crystal structure of Complex **2** was further stabilized by the weak interactions of intramolecular and intermolecular hydrogen bonds (Table S3). The oxygen atom (O1) from the phenolic hydroxyl group acted as the H-donor to the oxygen atom (O2(ii)) from the acetate, forming the hydrogen bond O1—H1…O2(ii) of 1.84 Å. By the intermolecular hydrogen bond, the asymmetric units of Complex **2** were linked in an infinite 1D hand-in-hand-like chain structure along the a axis. Meanwhile, the hydrogen bond C9—H9A…O3(iii) of 2.37 Å also existed between the oxygen atom (O3(iii)) from another acetate and the carbon atom(C9) from the ligand, extending the 1D hand-in-hand-like chain structure into a 2D wave-like network structure (Figure 5).

### 2.4. PXRD of Complexes ***1*** and ***2***

In order to check the phase purity and the crystallinity of the synthesized samples, powder X-ray diffraction experiments were carried out at room temperature. As shown in Figures S1 and S2, the main peak positions of the experimental spectra matched well with those of their simulated spectra from the corresponding single-crystal data, demonstrating the high phase purity and crystallinity of the samples. There were a few minor variations in the intensities and the widths of some peaks between the experimental and simulated spectra, which may be attributed to the superior orientation of the powder samples.

### 2.5. Catalytic Application on the Henry Reaction

#### 2.5.1. Optimization of Catalytic Reaction Conditions

Inspired by the promising applications in the field of organic synthesis, we investigated the catalytic activities of the synthesized complexes in the Henry reaction. After a series of abortive attempts, the desired products were obtained in relatively good yields. In order to further improve the catalytic system, the Henry reaction of nitromethane with benzaldehyde was chosen as the model reaction, and the reaction conditions such as catalyst loading, catalyst type, Et_3_N loading, and reaction solvent were investigated. The effect of various conditions on the Henry reaction is summarized in Table 1.

Considering the critical importance of the catalyst loading, we first explored the effect of catalyst loading on the reaction. The catalytic reaction could work well, producing the target product by performing the reaction in MeOH at room temperature in the presence of 10 to 14 mol% of Complex **1** with 20 mol% Et_3_N as the co-catalyst. As we expected, the yield increased with the increase of the catalyst loading within a certain range. When the catalyst loading increased to 10 mol%, the yield reached 81% (Entry 3). Continuing to increase the catalyst loading, the yield remained almost constant (Entries 4 and 5). Compared to Complex **1**, Complex **2** provided a 78% yield lower than that of Complex **1** (Entry 6), indicating its weaker catalytic activities. Based on the yield and catalytic cost, Complex **1** with the catalyst loading of 10 mol% was preferentially chosen as the catalyst. Additionally, the ligand was also used alone to catalyze the reaction, but no target product was obtained (Entry 9). Et_3_N showed poor catalytic activities in catalyzing the reaction, and only a 35% yield was obtained (Entry 10). Ni(OAc)_2_ × 4H_2_O and Zn(OAc)_2_ × 2H_2_O could also be used to catalyze the Henry reaction alone, but the yields were very low, only 19% and 15% (Entries 11 and 12). As an important co-catalyst, Et_3_N took part in the catalytic process and had a positive effect on the catalytic activities of the complexes. Without the addition of Et_3_N, Complexes **1** and **2** were used alone to catalyze the Henry reaction, and the yields decreased dramatically to only 35% and 33% (Entries 7 and 8), respectively. In view of the importance of Et_3_N, we turned our attention towards the research of the Et_3_N loading. When the Et_3_N loading was below 20 mol%, the catalytic activities of Complex **1** increased rapidly with the increase of the Et_3_N loading. Increasing the Et_3_N loading to 20 mol%, the yield reached 81% (Entry 3), while a further increase of the Et_3_N loading failed to enhance the catalytic yield (Entries 15 and 16). Therefore, Complex **1** with 20 mol% Et_3_N as the co-catalyst was selected as the optimum one. Moreover, there was a close relationship between the yield and the nature of the reaction solvent [32,33,34]. The reaction could be carried out in some aprotic solvents such as THF, CH_2_Cl_2_, and toluene, and only moderate yields were obtained (Entries 19–21). Compared with the aprotic solvents, some protic solvents such as methanol, isopropanol, and ethanol were effectively used to initiate the Henry reaction, and no less than a 76% yield was obtained (Entries 3, 17, and 18). In view of the higher yield, methanol rather than isopropanol and ethanol was chosen as the suitable solvent for the reaction.

#### 2.5.2. Expansion of the Substrate Scope on the Henry Reaction

In order to extend the substrate scope, we continued to intensify our efforts in the systematic research on the Henry reaction of nitromethane with various aromatic aldehydes (Table 2). In general, no matter whether the aromatic aldehydes contained electron-withdrawing or electron-donating substituent and contained ortho-, meta-, or para-substituent, some of them could smoothly undergo the Henry reaction in the presence of Complex **1** with 20 mol% Et_3_N as the co-catalyst. The β-nitroalcohol products with the yields in the range of 58–93% were obtained under the optimized conditions. However, it was also noted that the electronic effect and the steric hindrance of the substituent on the phenyl ring had some influence on the reaction. Aromatic aldehydes containing the electron-withdrawing substituent (Entries 4–8, 11–12) gave the corresponding products in high yields, while those containing the electron-donating substituent (Entries 2–3, 9–10) were converted into the catalytic products in relatively low yields. The main reason lied in the fact that the electron-withdrawing substituent of aromatic aldehydes increased the electropositive character of carbonyl carbon, which was beneficial to the nucleophilic addition reaction of nitromethane with aromatic aldehydes. In addition, influenced by the steric hindrance of the substituent, the different yields were obtained by the reaction of nitromethane with aromatic aldehydes with the ortho-, meta-, or para-substituent at the phenyl ring, indicating their different catalytic activities. Compared with some other complexes reported [35,36], Complex **1** had slight advantages in terms of the catalytic yield of the Henry reaction of nitromethane with some aromatic aldehydes.

#### 2.5.3. Mechanistic Investigation of the Henry Reaction

**Figure 6:**
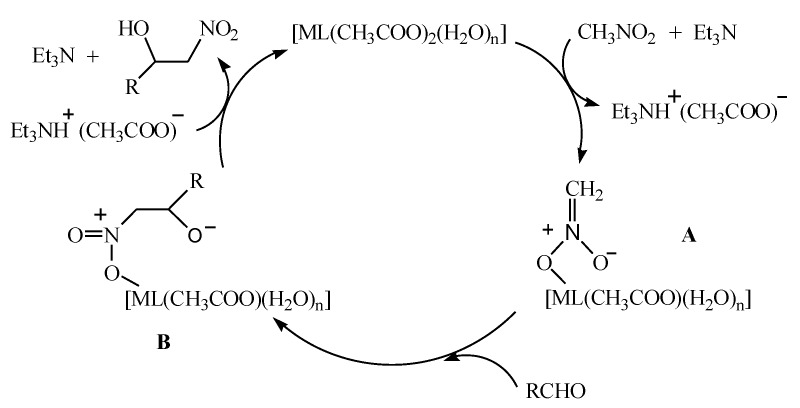
The possible mechanism of the Henry reaction.

According to the above experimental results and the reported literature [37,38,39], we managed to deduce the possible reaction mechanism of nitromethane with aromatic aldehydes in the presence of [ML(CH_3_COO)_2_(H_2_O)_n_] (n = 1, M = Ni; n = 0, M = Zn) with 20 mol% Et_3_N as the co-catalyst. As shown in Figure 6, under the action of alkaline triethylamine, nitromethane was activated to coordinate to the bivalent metal cation in [ML(CH_3_COO)_2_(H_2_O)_n_], presenting a metal complex intermediate A and a triethylammonium acetate. Simultaneously, the nucleophilic reaction between the intermediate A and aromatic aldehyde was carried out, resulting in the formation of a complex intermediate B. The intermediate B then reacted with the triethylammonium acetate generated earlier to obtain the corresponding β-nitroalcohol product, and at the same time, a bivalent metal complex and a triethylamine molecule were released.

## 3. Materials and Methods

### 3.1. General

All aromatic aldehydes were of analytical purity and were purchased from Shanghai Aladdin Industrial Co., Ltd. (Shanghai, China). Analytical grade diethylenetriamine, nitromethane, and salicylaldehyde were purchased from Alfa Aesar China Co. Ltd. (Beijing, China). Other reagents, such as Ni(OAc)_2_ × 4H_2_O, Zn(OAc)_2_ × 2H_2_O, and NaBH_4_, were obtained from Sinopharm Chemical Reagent Co., Ltd. (Shanghai, China).

FTIR spectra of Complexes **1** and **2** were conducted with an FTS-40 spectrometer (Bio-Rad, Santa Clara, CA, USA) in the region 400–4000 cm^−1^ with KBr pellets. NMR spectra were performed on a Bruker AMX-400 spectrometer (Bruker, Billerica, MA, USA) in CDCl_3_ solution with TMS as the internal standard. Powder X-ray diffractions (PXRD) were performed on a DX-2600 diffractometer (Dandong, Liaoning, China) with Cu Kα radiation at ambient temperature. Elemental analyses (C, H, and N) for the complexes and the ligand were carried out on a Vario EL III elemental analyzer (Elementar, Bremen, Germany).

### 3.2. Synthesis

#### 3.2.1. Synthesis of the Ligand

To a water solution (30 mL) of diethylenetriamine (2.58 mL, 23.95 mmol) was added salicylaldehyde (5.00 mL, 47.90 mmol). The resulting solution was stirred at room temperature for 1.5 h. The yellow organic layer was separated from the reaction solution and washed with n-hexane (2 × 10 mL). The crude product was directly used for the following oxidation-reduction reaction. NaBH_4_ (1.81 g, 47.90 mmol) was added to a methanol solution (40 mL) of the product in three equal lots, and the resulting solution was stirred for another 4 h. Upon adding water (30 mL), the solution was fully extracted with dichloromethane (3 × 20 mL). The organic layer separated from the water layer was dried over anhydrous MgSO_4_, filtered, and concentrated in vacuo to yield a colorless oily product. Yield: 83%. Anal. Calcd. for C_18_H_25_N_3_O_2_ (MW = 315.41): C, 68.54; H, 7.99; N, 13.32%. Found: C, 68.35; H, 8.23; N, 13.29%. ^1^H-NMR (400MHz, CDCl_3_): δ = 7.16–6.75 (m, 8H, Ar-H), 4.02 (s, 4H, Ar-CH_2_-N), 3.31–3.23 (m, 8H, CH_2_CH_2_). ^13^C-NMR (100MHz, CDCl_3_): δ = 160.2, 129.6, 129.1, 118.6, 118.3, 116.7, 60.4, 53.3, 47.7.

#### 3.2.2. Synthesis of [NiL(CH_3_COO)_2_(H_2_O)] (**1**)

To an EtOH solution (30 mL) of Ni(OAc)_2_·4H_2_O (0.79 g, 3.17 mmol) was added an EtOH solution (10 mL) of the ligand (1.00 g, 3.17 mmol). The reaction mixture was heated to reflux for 36 h with stirring at 351 K. The final solution was filtered, and the filtrate was evaporated in a vacuum to provide a dark green residue. After the solution was left standing for 72 h under room temperature, block-like crystals of Complex **1** were obtained by recrystallization with a co-solvent of methanol and dichloromethane (*v*/*v* = 2/1). Yield: 64%, m.p. 201.5–203.5 °C. IR (KBr, cm^−1^): 3567(s), 3459(vs), 2716(m), 2631(m), 1596(s), 1552(vs), 1405(vs), 1263(m), 1241(s), 1187(w), 1154(m), 1115(m), 908(w), 848(m), 744(m), 706(m), 679(m), 619(w), 495(w). Anal. Calcd. for C_22_H_33_N_3_NiO_7_ (MW = 510.22): C, 51.79; H, 6.52; N, 8.24%. Found: C, 51.65; H, 6.80; N, 7.95%.

#### 3.2.3. Synthesis of [ZnL(CH_3_COO)_2_] (**2**)

The preparation of Complex **2** was analogous to that of Complex **1**, except that Ni(OAc)_2_ × 4H_2_O was replaced by Zn(OAc)_2_ × 2H_2_O (0.70 g, 3.17 mmol). Colourless needle-like crystals of Complex **2** were obtained by recrystallization with a co-solvent of ethanol and dichloromethane (*v*/*v* = 1/1). Yield: 72%, m.p. 185.0–186.5 °C. IR (KBr, cm^−1^): 3367(s), 3069(vs), 2729(s), 2613(m), 1925(w), 1574(s), 1424(s), 1328(m), 1320(w), 1268(s), 1247(s), 1187(m), 1151(m), 1064(m), 1014(w), 984(m), 911(w), 867(m), 822(w), 747(s), 676(s), 623(m), 541(m). Anal. Calcd. for C_22_H_31_N_3_O_6_Zn (MW = 498.87): C, 52.96; H, 6.26; N, 8.42%. Found: C, 53.05; H, 5.99; N, 8.25%.

### 3.3. Single-Crystal X-ray Diffraction

Diffraction data collection of [NiL(CH_3_COO)_2_(H_2_O)] and [ZnL(CH_3_COO)_2_] was performed on a Bruker APEX-II CCD diffractometer (Bruker, Karlsruhe, Germany) at 296(2) K, using an *φ~ω* scan mode and graphite-monochromated Mo Kα radiation (λ = 0.71073 Å). The crystal structures were solved by direct methods with the SHELXT program [39,40] and refined by the full-matrix least-squares method based on F^2^ with the SHELXL-2018/3 program [41]. All hydrogen atoms were generated geometrically and refined isotropically by using a riding model, while other atoms in the structures were refined with anisotropic displacement parameters. In the asymmetric unit of Complex **2**, two carbon atoms of the diethylenetriamine group from the ligand were found to be disordered in two different orientations and refined by splitting the moiety over two parts. Further information about crystallographic data for Complexes **1** and **2** is summarized in Table 3, and the selected bond lengths and angles are listed in Table S2.

### 3.4. General Procedure for the Henry Reaction

To a methanol solution (10 mL) of the synthesized complex (0.10 mmol) was added triethylamine (0.20 mmol). Then, the appropriate aromatic aldehyde (1.00 mmol) and nitromethane (6.00 mmol) were added to the resulting clear solution via a syringe. The resulting mixture was stirred at room temperature for 18 h, and the extent of the reaction was monitored by TLC. After evaporation of the volatile components, the residue was isolated by column chromatography with n-hexane and ethyl acetate (*v*/*v* = 5/1) as the eluent, and the corresponding β-nitroalcohol product was obtained. The molecular structures of the products were characterized by ^1^H-NMR and ^13^C-NMR (Table S1).

## 4. Conclusions

In summary, two mononuclear zero-dimensional complexes, namely [NiL(CH_3_COO)_2_(H_2_O)] and [ZnL(CH_3_COO)_2_], were synthesized by the reactions of N, N’-bis(2-hydroxybenzyl)diethylenetriamine to Ni(OAc)_2_ × 4H_2_O and Zn(OAc)_2_ × 2H_2_O, respectively. The asymmetric unit of Complex **1** consisted of two acetate anions, one ligand, one water molecule, and one Ni(II) cation. The asymmetric unit of Complex **2** consisted of one acetate, half of Zn(II) cation, and half of the ligand. By their respective hydrogen bonds, the asymmetric units of Complex **1** were further linked into an infinite 1D ladder-like chain structure, and the asymmetric units of Complex **2** were further linked into a 2D wave-like network structure.

Furthermore, the complexes were found to be efficient and practical catalysts for the Henry reaction. The optimized conditions were obtained as follows: catalyst loading of 10 mol%, in methanol, and in the presence of Complex **1** with 20 mol% Et_3_N as the co-catalyst. Further investigation indicated that the reaction could be carried out between various aromatic aldehydes and nitromethane, showing a broad substrate applicability. Moreover, the possible catalytic mechanism of the Henry reaction was also deduced.

## Figures and Tables

**Table 1:**
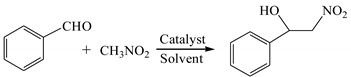
Effect of different conditions on the catalytic reaction ^a^.

Entry	Catalyst Loading ^b^ (mol%)	Catalyst	Et_3_N Loading ^c^ (mol%)	Solvent	Yield (%)
1	6	**1** + Et_3_N	20	MeOH	39
2	8	**1** + Et_3_N	20	MeOH	68
3	10	**1** + Et_3_N	20	MeOH	81
4	12	**1** + Et_3_N	20	MeOH	81
5	14	**1** + Et_3_N	20	MeOH	82
6	10	**1** + Et_3_N	20	MeOH	78
7	10	**1**	20	MeOH	35
8	10	**2**	20	MeOH	33
9	10	L	20	MeOH	0
10	10	Et_3_N	20	MeOH	35
11	10	Ni(OAc)_2_ × 4H_2_O	0	MeOH	19
12	10	Zn(OAc)_2_ × 2H_2_O	0	MeOH	15
13	10	**1** + Et_3_N	10	MeOH	56
14	10	**1** + Et_3_N	15	MeOH	73
15	10	**1** + Et_3_N	25	MeOH	81
16	10	**1** + Et_3_N	30	MeOH	81
17	10	**1** + Et_3_N	20	*^i^*PrOH	79
18	10	**1** + Et_3_N	20	EtOH	76
19	10	**1** + Et_3_N	20	THF	71
20	10	**1** + Et_3_N	20	CH_2_Cl_2_	62
21	10	**1** + Et_3_N	20	Toluene	66

^a^ Henry reaction: nitromethane 6.00 mmol, benzaldehyde 1.00 mmol, complex 0.10 mmol, Et_3_N 0.20 mmol, solvent 10 mL, room temperature, reaction time 18 h; ^b^ catalyst loading (mol%): complex/benzaldehyde; ^c^ Et_3_N loading (mol%): Et_3_N/benzaldehyde.

**Table 2:**
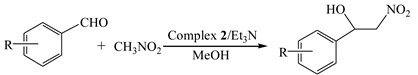
Henry reaction of different aromatic aldehydes with nitromethane.

Entry	Compound	R	Yield (%)
1	**3**	H	81
2	**4**	*p*-Me	72
3	**5**	*o*-Me	77
4	**6**	*p*-Cl	90
5	**7**	*o*-Cl	87
6	**8**	*m*-Cl	83
7	**9**	*p*-Br	85
8	**10**	*m*-Br	82
9	**11**	*p*-MeO	66
10	**12**	*o*-MeO	58
11	**13**	*p*-NO_2_	93
12	**14**	*m*-NO_2_	88

**Table 3:** Crystallographic data for Complexes **1** and **2.**

Complex	1	2
Formula	C_22_H_33_N_3_NiO_7_	C_22_H_31_N_3_O_6_Zn
Fw	510.22	498.87
Crystal system	Monoclinic	Monoclinic
Space group	*P*2_1_/*n*	*C*_2_/*c*
*a* (Å)	8.687 (5)	16.798 (4)
*b* (Å)	15.248 (8)	6.5119 (17)
*c* (Å)	20.858 (11)	22.933 (6)
*α* (◦)	90	90
*β* (◦)	93.384 (10)	106.792 (9)
*γ* (◦)	90	90
Volume (Å^3^)	2758 (3)	2401.6 (11)
Z	4	4
Dcalcd/(g·cm^−3^)	1.229	1.38
*θ* range (◦)	1.7–27.3	2.7–27.4
*μ* (mm^−1^)	0.744	1.064
*F* (000)	1080	1048
Crystal size (mm)	0.12 × 0.13 × 0.14	0.22 × 0.24 × 0.26
Reflections collected	15,493	10,092
Unique reflections (R_int_)	5971 (0.050)	2740 (0.042)
Goodness-of-fit on F^2^	1.014	1.031
Final *R* indices [I > 2σ(I)]	*R*_1_ = 0.0509, *wR*_2_ = 0.1195	*R*_1_ = 0.0376, *wR*_2_ = 0.0890
*R* indices (all data)	*R*_1_ = 0.0936,*wR*_2_ = 0.1351	*R*_1_ = 0.0500, *wR*_2_ = 0.0945
Largest diff. peak and hole (e Å^−3^)	0.55 and −0.38	0.29 and −0.35

^a^ R = Σ(||F_0_| − F_c_||)/Σ|F_0_|, ^b^ wR = [Σw(|F_0_|^2^ − |F_c_|^2^)^2^/Σw(F_0_^2^)]^1/2^.

## Supplementary Materials

The following are available online: Table S1. ^1^H-NMR and ^13^C-NMR characterization of the catalytic products; Table S2. Selected bond lengths (Å) and angles (*°*) of Complexes **1** and **2**; Table S3. Hydrogen bonding parameters of Complexes **1** and **2**; Figure S1. PXRD patterns of Complex **1**; Figure S2. PXRD patterns of Complex **2**. CCDC-1989226 for Complex **1** and CCDC-1989228 for Complex **2**.
