# Supplementary material for: Synthesis and Catalytic Application of Two Mononuclear Complexes Bearing Diethylenetriamine Derivative Ligand
Source: Molecules. 2020 Apr 30;25(9):2101. doi: 10.3390/molecules25092101 (PMC7248821; doi:10.3390/molecules25092101)
Supplement: Supplementary file 1 [file molecules-25-02101-s001.pdf]

Synthesis and Catalytic Application of Two Mononuclear Complexes Bearing Diethylenetriamine Derivative Ligand

Chao Liu*, Zhao Yang, Hao Guo and Yu-Cai Zhao

School of Chemistry and Chemical Engineering, Suzhou University, Suzhou 234000, China; hgyangzhao@stu.ahszu.edu.cn (Z.Y.); hgguohao@stu.ahszu.edu.cn (H.G.); zhoufengmei@ahszu.edu.cn (Y.-C.Z.)

* Correspondence: kykrliu@ahszu.edu.cn (C.L.); Tel.: +86-557-2875018

Supplementary materials

Table S1. ^{1}H NMR and ^{13}C NMR characterization of the catalytic products.

Table S2. Selected bond lengths (Å) and angles (º) of complexes **1** and **2**.

Table S3. Hydrogen bonding parameters of complexes **1** and **2**.

Figure S1. PXRD patterns of complex **1**.

Figure S2. PXRD patterns of complex **2**.

Table S1. ^{1}H NMR and ^{13}C NMR characterization of the catalytic products.

Product	NMR
**3**	δ_H: 7.41-7.29 (m, 5H, Ar-H), 5.43 (dd, *J* = 3.0, 9.6 Hz, 1H, ArCH), 4.58 (dd, *J* = 9.6, 13.0 Hz, 1H, CH_2), 4.46 (dd, *J* = 3.0, 13.0 Hz, 1H, CH_2), 2.84 (br, 1H, OH). δ_C: 138.1, 128.9, 128.8, 125.8, 81.2, 70.9.
**4**	δ_H: 7.52-7.36 (m, 4H, Ar-H), 5.45 (dd, *J* = 3.2, 9.2 Hz, 1H, ArCH), 4.58 (dd, *J* = 9.4, 13.2 Hz, 1H, CH_2), 4.44 (dd, *J* = 3.2, 13.2 Hz, 1H, CH_2), 3.39 (br, 1H, OH), 2.37 (s, 3H, CH_3). δ_C: 138.7, 135.0, 129.6, 125.6, 81.1, 70.7, 21.0.
**5**	δ_H: 7.48-7.15 (m, 4H, Ar-H), 5.63 (dd, *J* = 2.9, 9.4 Hz, 1H, ArCH), 4.50 (dd, *J* = 9.4, 13.4 Hz, 1H, CH_2), 4.41 (dd, *J* = 2.9, 13.4 Hz, 1H, CH_2), 2.99 (br, 1H, OH), 2.26 (s, 3H, CH_3). δ_C: 136.1, 134.4, 130.8, 128.6, 126.8, 125.6, 80.3, 68.1, 21.2.
**6**	δ_H: 7.41-7.28 (m, 4H, Ar-H), 5.45 (dd, *J* = 3.4, 9.8 Hz, 1H, ArCH), 4.59 (dd, *J* = 9.8, 13.0 Hz, 1H, CH_2), 4.50 (dd, *J* = 3.4, 13.0 Hz, 1H, CH_2), 3.05 (br, 1H, OH). δ_C: 136.6, 134.8, 129.2, 127.4, 81.1, 70.3.
**7**	δ_H: 7.55-7.23 (m, 4H, Ar-H), 5.83 (dd, *J* = 3.2, 9.8 Hz, 1H), 4.58 (dd, 1H, *J* = 9.6, 13.2Hz), 4.38(dd, 1H, *J* = 3.2, 13.2 Hz), 3.12 (br, 1H, OH). δ_C: 135.6, 131.4, 130.0, 129.7, 127.7, 127.5, 79.3, 67.9.
**8**	δ_H: 7.38-7.20 (m, 4H, Ar-H), 5.38 (dd, *J* = 3.2, 9.6 Hz, 1H, ArCH), 4.53 (dd, 1H, *J* = 9.6, 13.5 Hz, 1H, CH_2), 4.46 (dd, 1H, *J* = 3.2, 13.5 Hz, 1H, CH_2), 3.54 (br, 1H, OH). δ_C: 140.1, 135.1, 130.5, 139.1, 126.2, 124.1, 81.0, 70.3.
**9**	δ_H: 7.45-7.22 (m, 4H, Ar-H), 5.43 (dd, 1H, *J* = 2.6, 9.4 Hz, 1H, ArCH), 4.55 (dd, *J* = 9.4, 13.2 Hz, 1H, CH_2), 4.47 (dd, *J* = 2.6, 13.2 Hz, 1H, CH_2), 3.25 (br, 1H, OH). δ_C: 136.9, 132.1, 127.5, 122.8, 80.8, 70.3.
**10**	δ_H: 7.39-7.20 (m, 4H, Ar-H), 5.42 (dd, *J* = 3.2, 9.2 Hz, 1H, ArCH), 4.59 (dd, *J* = 9.2, 13.6 Hz, 1H,CH_2), 4.48 (dd, *J* = 3.2, 13.6 Hz, 1H, CH_2), 3.59 (br, 1H, OH). δ_C: 140.2, 132.1, 130.6, 129.0, 124.6, 123.3, 81.0, 70.3.
**11**	δ_H: 7.44-6.91 (m, 4H, Ar-H), 5.61 (dd, *J* = 3.4, 9.6 Hz, 1H, ArCH), 4.59 (dd, *J* = 9.6, 13.4 Hz, 1H, CH_2), 4.48 (dd, *J* = 3.4, 13.4 Hz, 1H, CH_2), 3.85 (s, 3H, OCH_3), 3.03 (br, 1H, OH). δ_C: 159.5, 130.0, 126.8, 113.9, 80.9, 70.2, 54.9.
**12**	δ_H: 7.46-7.21 (m, 4H, Ar-H), 5.60 (dd, *J* = 3.2, 9.8 Hz, 1H, ArCH), 4.66 (dd, *J* = 9.8, 13.6 Hz, 1H, CH_2), 4.57 (dd, *J* = 3.0, 13.6 Hz, 1H, CH_2), 3.87 (s, 3H, OCH_3), 3.11 (br, 1H, OH). δ_C: 155.8, 129.7, 127.1, 125.9, 121.0, 110.5, 79.7, 67.6, 55.3.
**13**	δ_H: 8.34-7.60 (m, 4H, Ar-H), 5.63 (dd, *J* = 3.2, 8.2 Hz, 1H, ArCH), 4.61 (dd, *J* = 8.2, 13.4 Hz, 1H, CH_2), 4.56 (dd, *J* = 3.2, 13.4 Hz, 1H, CH_2), 3.35 (br, 1H, OH). δ_C: 148.0, 144.7, 126.8, 124.0, 80.5, 69.8.
**14**	δ_H: 8.32-7.60 (m, 4H, Ar-H), 5.60 (dd, *J* = 3.2, 8.4 Hz, 1H, ArCH), 4.65 (dd, *J* = 8.4, 13.2 Hz, 1H, CH_2),4.58 (dd, *J* = 3.2, 13.2 Hz, 1H, CH_2), 3.27 (br, 1H, OH). δ_C: 148.5, 140.1, 131.9, 129.9, 123.7, 121.1, 80.5, 69.7.

Table S2. Selected bond lengths (Å) and angles (º) of complexes **1** and **2**.

**Complex 1**							
Ni1—O3	2.074(2)	Ni1—O5	2.031(3)	Ni1—O7	2.175(2)	Ni1—N1	2.114(3)
Ni1—N2	2.065(3)	Ni1—N3	2.121(3)				
O3—Ni1—O5	96.60(9)	O3—Ni1—O7	173.65(10)	O3—Ni1—N1	93.75(9)	O3—Ni1—N2	84.88(9)
O3—Ni1—N3	93.65(9)	O5—Ni1—O7	88.96(9)	O5—Ni1—N1	97.95(10)	O5—Ni1—N2	177.54(8)
O5—Ni1—N3	94.63(10)	O7—Ni1—N1	82.40(9)	O7—Ni1—N2	89.67(9)	O7—Ni1—N3	88.95(9)
N1—Ni1—N2	83.89(10)	N1—Ni1—N3	164.56(11)	N2—Ni1—N3	83.30(10)		
**Complex 2**							
Zn1—O3	1.974(2)	Zn1—N1	2.198(2)	Zn1—N2	2.092(3)	Zn1—O3(i)	1.974(2)
Zn1—N1(i)	2.198(2)						
O3—Zn1—N1	94.68(8)	O3—Zn1—N2	129.73(6)	O3—Zn1—O3(i)	100.55(9)	O3—Zn1—N1(i)	96.96(8)
N1—Zn1—N2	80.87(5)	O3(i)—Zn1—N1	96.96(8)	N1—Zn1—N1(i)	161.74(7)	O3(i)—Zn1—N2	129.73(6)
N1(i)—Zn1—N2	80.87(5)	O3(i)—Zn1—N1(i)	94.68(8)				

Symmetry code: (*i*) 1-x,y,1/2-z for complex **2**.

Table S3. Hydrogen bonding parameters of complexes **1** and **2**.

Complex	D—H···A	D—H (Å)	H···A(Å)	D···A(Å)	D—H···A(º)
**1**	N1—H1A···O1	0.98	2.57	3.173(4)	119
	O2—H2···O6(i)	0.82	1.82	2.642(4)	178
	N3—H3···O2	0.98	2.43	3.058(4)	121
	O7—H7A···O4(ii)	0.77	1.97	2.731(3)	173
	O7—H7B···O6	0.82	1.79	2.612(4)	175
	C19—H19C···O5	0.96	2.45	3.142(4)	129
**2**	O1—H1···O2(ii)	0.82	1.84	2.655(3)	171
	N1—H1···O1	0.98	2.40	3.022(3)	121
	N1—H1A···O2	0.98	2.57	3.152(3)	118
	C9—H9A···O3(iii)	0.97	2.37	3.145(6)	137

Symmetry codes: (i) 1-x,1-y,1-z; (ii) 1+x,y,z for complex **1**; (ii) 1/2-x,-1/2+y,1/2-z; (iii) 1-x,1+y,1/2-z for complex **2**.

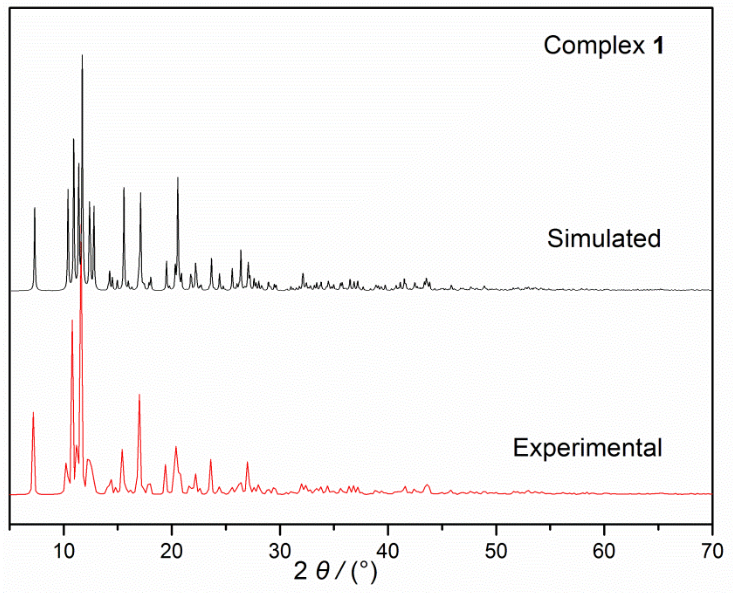

Figure S1. PXRD patterns of complex **1**.

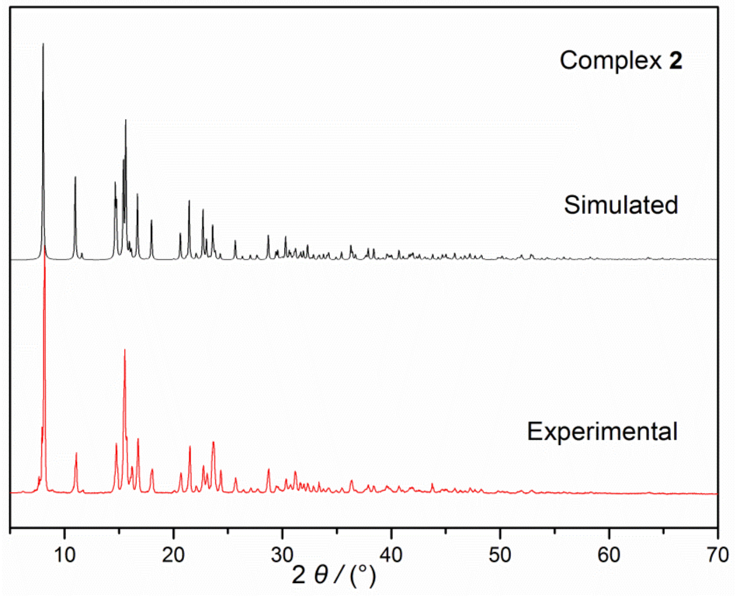

Figure S2. PXRD patterns of complex **2**.
